# Supplementary material for: Arabidopsis Restricts Sugar Loss to a Colonizing Trichoderma harzianum Strain by Downregulating SWEET11 and -12 and Upregulation of SUC1 and SWEET2 in the Roots
Source: Microorganisms. 2021 Jun 8;9(6):1246. doi: 10.3390/microorganisms9061246 (PMC8227074; doi:10.3390/microorganisms9061246)
Supplement: Supplementary file 1 [file microorganisms-09-01246-s001.zip › microorganisms-1245330-SI.pdf]

Figure S1: Acid phosphatase activity

Acid phosphatase activity (AcP) of *Trichoderma* spp in comparison to three control bacterial strains (top). Spectrophotometric determination of available phosphate concentration by *Trichoderma* spp in comparison to three control bacterial strains (bottom). Based on 3 independent experiments; bars represent SEs.

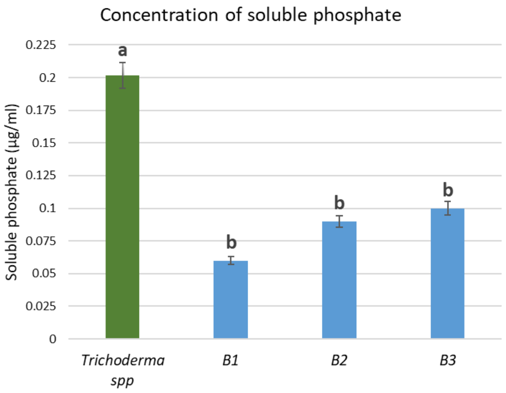

Figure S2: *SUC2, STP1, STP13, ERD6* and *ERD6-like6* in the roots of *Arabidopsis* seedlings

Relative mRNA levels for sugar transporter genes *SUC2*, *STP1*, *STP13*, *ERD6* and *ERD6-like6* in the roots of *Arabidopsis* seedlings after transfer to NBRIP medium for 48 h or 96 h. Upon transfer to the NBRIP medium, the seedlings were either co-cultured with *Trichoderma* (grey) or mock-treated (white). The mRNA levels are expressed relative to the levels in the roots at the time point of transfer to NBRIP medium (t = 0 h). Based on 3 independent experiments; bars represent SEs.

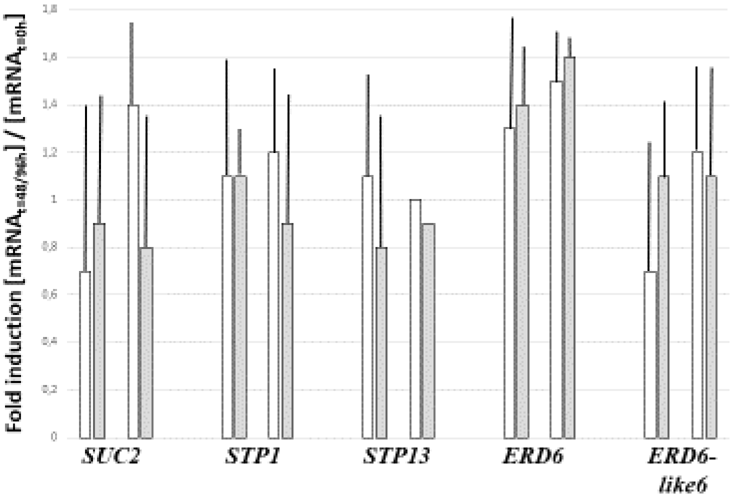

Table S1: Primer pairs

The primer pairs used for the study.

**Gene**	**forward**	**reverse**
PHO1	AGACAACCGGGTGTTACTTCTTGG	GTCCCACCATCTCCTTACACATTG
WRKY6	GTTGTTTCCTTCGCCGTCGTGG	ATGGACAGAGGATGGTCTGGTC
Pht1;1	CCTTTGGGTTCCTATATGCG	TAACCTCAGCCTCACCAGAG
Pht1;4	TGTGCCGGCCGAAATCT	TTGCTCCTAATTTTCCTGATGCT
PHR1	AAACCAACCCGGCGATTCA	CAGCCCATTCATGCCAATCACTT
PDF1.2	CGCACCGGCAATGGTGG	ATCCATGTTTGGCTCCTTCG
PR1	GTAGGTGCTCTTGTTCTTCCC	CACATAATTCCCACGAGGATC
ZEP	GATGCAGCCAAATATGGGTCAAGG	GCCATTGCATGGATAATAGCGACTC
SUC1	GACCTTTCGACGCCTTGTTC	AATACTCCACTAATCGCCGCTG
SUC2	GACCTTTCGACGCCTTGTTC	AATACTCCACTAATCGCCGCTG
STP1	TGGAGGATGGTGAGTATGG	TGGTTACTGTTCTTGCCCATCT
STP13	TATGGGACCGCCAAGATTAAA	AAGCTCCGACCGTTAGAAGAA
ERD6	ATAATGGCTGAGATATTTCCG	ATAAATACGATCGAACTGGC
ERD6-like6	GTGGAGGAACTTTCACTCTGTA	GTTCTTCAAGAGTTTTGCCTT
SWEET2	CACGGTGGTACTTTGTCGGG	AACGGCATAAACTCAACGTCT
SWEET3	GAGTCGGCATCCTTCTCGAA	ACCAAGGCTGAGATTGCTGTC
SWEET11	GCCAATCTCAGTGGTTCGTCAAG	GAAGAGGACTGCTTGCCATGT
SWEET12	CTCACATCTCCTGAACCAGTAGC	TGCAGCACTGTTTCTAACTCCC
UBIQUITIN5	GACGCTTCATCTCGTCC	GTAAACGTAGGTGAGTCCA
ACTIN2	TCCAAGCTGTTCTCTCCTTG	GAGGGCTGGAACAAGACTTC
tef1	TCAAGTCCGTTGAGATGCAC	CGTTCTTGACGTTGAAACCA
